# A phase I study of ridaforolimus in adult Chinese patients with advanced solid tumors

**DOI:** 10.1186/1756-8722-6-48

**Published:** 2013-07-08

**Authors:** Lian Liu, Wen Zhang, Wenhua Li, Fangfang Lv, Zuguang Xia, Sheng Zhang, Wen Liu, Anthe S Zandvliet, Sylvia Waajen, Li Xin Zhang, Li Yan, Jin Li

**Affiliations:** 1 Department of Medical Oncology, Fudan University Shanghai Cancer Center, Shanghai, China; 2 Department of Oncology, Shanghai Medical College, Fudan University, Shanghai, China; 3 Merck Research Laboratory, Merck and Co., Inc., Pennsylvania, USA

**Keywords:** MTOR inhibitor, Phase I study, Pharmacokinetic analysis, Adverse event

## Abstract

**Purpose:**

Ridaforolimus (AP23573, MK-8669 or deforolimus) is an inhibitor of mammalian target of rapamycin (mTOR), an important regulator in the cell survival pathway. This open-label, single center phase I study aimed to investigate the pharmacokinetic (PK) and safety profiles of ridaforolimus in Chinese patients with treatment-refractory advanced or relapsed solid tumors. The PK data generated from these Chinese patients were further compared with those previously reported in Caucasian and Japanese patient populations.

**Experimental design:**

The patients were given an oral dose of 40 mg of ridaforolimus on Day 1 of the study. On Day 8, patients were initiated on a treatment regimen that comprised a once daily dose of 40 mg of ridaforolimus for five consecutive days, followed by a 2-day off-drug interval. Patients repeated this regimen until disease progression or intolerance. Blood samples were collected at specific times pre- and post-treatment to establish the PK profile of ridaforolimus in all patients.

**Results:**

Fifteen patients were given at least one dose of 40 mg of ridaforolimus. The median absorption lag-time was 2 hours, the median T_max_ was 4 hours and the mean elimination half-life was 53 hours. The accumulation ratio for AUC_0-24hr_ was 1.3 on day 19 (steady state)/day 1 (after a single dose). The most common drug-related adverse events (AEs) that occurred in ≥40% of patients were stomatitis, proteinuria, leukopenia, hyperglycemia, and pyrexia. Grade 3/4 drug-related AEs were anemia, stomatitis, fatigue, thrombocytopenia, constipation, gamma glutamyltransferase increase, and proteinuria. All 11 evaluable patients achieved stable disease.

**Conclusions:**

Oral ridaforolimus at a daily dose of 40 mg were generally well tolerated in Chinese patients with advanced or refractory solid tumors. Adverse events and PK profiles of ridaforolimus in this study were similar to those from Caucasian and Japanese patients reported previously.

## Introduction

Mammalian target of rapamycin is a serine/threonine kinase that fulfills a pivotal role in regulating cell growth and proliferation. Together with the phosphatidylinositol-3-kinase (PI3K)-AKT transduction pathway, mTOR forms a signaling network that has been implicated in various types of cancer [1]. This signaling network is considered a validated target for innovative cancer therapy and therefore mTOR inhibitors are thought to have potent antitumor activity [2-5]. Active signaling via mTOR requires formation of the ternary complex mTOR Complex-1 and mTOR Complex-2. Activity of mTOR complex-1 can be inhibited by rapamycin and several rapamycin analogs have been developed to serve as potential antitumor agents.

Ridaforolimus (Merck & Co., Inc. Whitehouse Station, NJ, USA) is a non-prodrug analog of rapamycin and pre-clinical studies showed its anti-tumor activity in a wide variety of cancers [6]. Furthermore, phase II clinical trials with ridaforolimus as monotherapy showed good tolerability as well as clinical activity in prostate cancer [7], in bone and soft tissue sarcomas [8], and in hematologic malignancies [9]. In a phase III trial, as maintenance therapy, ridaforolimus significantly improved progression free survival (PFS) when compared with a placebo in patients with advanced sarcoma who had clinical benefit from prior standard cytotoxic chemotherapy (P = 0.0001; median PFS: 17.7 weeks vs 14.6 weeks for ridaforolimus and a placebo, respectively). Median overall survival at data cut-off (386 deaths), was 93.3 weeks in patients given ridaforolimus vs 83.4 weeks in patients given a placebo (P = 0.23) [10].

The safety, tolerability, and pharmacokinetics (PK) of oral ridaforolimus have been investigated previously in Caucasians and Japanese patients, the most important dose limiting toxicity is stomatitis, and the oral dose of 40mg per day for five consecutive days followed by two day's off was well tolerated [11-14]. As PK could be different between Chinese population and Japanese population, this phase I trial was designed to evaluate the safety, tolerability and PK of oral ridaforolimus in Chinese patients with treatment-refractory advanced or relapsed solid tumors.

## Methods

### Patients

Chinese patients older than 18 years of age with histologically or cytologically proven metastatic or locally advanced solid tumor(s), who progressed on standard therapy, or for whom standard therapy does not exist, were eligible for enrolment. Inclusion criteria also included Eastern Cooperative Oncology Group (ECOG) performance status of ≤1, acceptable hematologic, hepatic, and renal function with absolute neutrophil count ≥1,500/μL, platelet count ≥100,000/μL, hemoglobin level ≥9.0 g/dL, serum creatinine level ≤1.5 mg/dL or creatinine clearance ≥60 mL/min, total bilirubin level within normal range, aspartate aminotransferase and alanine aminotransferase level ≤2.5 × upper limits of normal, total cholesterol ≤350 mg/dL, triglycerides ≤400 mg/dL, and hemoglobin A1C < 8%. Patients also had to have a life expectancy of more than three months.

Exclusion criteria included chemo-, radio-, or biological-therapy within four weeks prior to enrolment; primary or unstable central nervous system metastasis; clinically significant abnormality on electrocardiogram; and New York Heart Association Class III or IV congestive heart failure or any other significant history of cardiac disease.

### Ethics

The study was approved by the institutional review board of the Fudan University Shanghai Cancer Center Ethics Committee and conducted in accordance with the Declaration of Helsinki and Good Clinical Practice. All patients provided written informed consent.

### Study design

This was an open-label, single-center PK study of ridaforolimus (clinicaltrials.gov registration, NCT01380184). The treatment dose of 40 mg of ridaforolimus per day was based on the results of previous phase I clinical studies that investigated various dosing schedules for ridaforolimus in non-Chinese patients (internal data).

The eligible patients were given the following treatment regimen: a single oral dose of 40 mg of ridaforolimus on Day 1; and then, the treatment started from Day 8 with an oral dose of 40 mg of ridaforolimus for five consecutive days (on Day 8 through Day 12) followed by two day's off (Day 13 and 14). The treatment repeated every 7 days. Four treatment weeks was considered one treatment cycle. Sample blood for PK analysis were collected at the following times: on Day 1 prior to drug administration and for 168 h post-dose (at 1, 2, 3, 4, 6, 8, 24, 72, 96, and 168 h post-treatment); on Day 8 and Day 15 prior to drug administration; on Day 19 prior to drug administration and for 24 h post-dose (at 1, 2, 3, 4, 6, 8 and 24 h post-treatment). We monitored patients’ vital signs and conducted laboratory and physical examinations on trial Day 1, 8 and 19.

The patients continued to receive 40 mg of ridaforolimus for five consecutive days followed by two day's off until disease progression or intolerability. Averse events were monitored every four weeks and AEs were graded according to the Common Terminology Criteria for Adverse Events version 4.0. We also monitored patients’ vital signs and conducted laboratory and physical examinations every four weeks. Radiographic studies for disease assessment were conducted at baseline and again after every two treatment cycles (thus, every eight weeks). Tumor responses were assessed according to the Response Evaluation Criteria for Solid Tumors 1.1 guidelines. We conducted a full safety assessment in all patients four weeks after the administration of the last treatment dose.

### Pharmacokinetic assessment

Ridaforolimus concentration in both whole blood and plasma was analyzed using high-performance liquid chromatography with tandem mass spectrometric detection set at a lower limit of quantification of 0.2 ng/mL (Wuxi AppTec, Shanghai, China). Pharmacokinetic analyses were performed with WinNonlin, version 5.2.1 (Pharsight Corp., St. Louis, MO, USA). AUC_0-∞_, AUC_0-24hr_, C_24hr_, and C_max_ were determined for each patient on Day 1 and on Day 19, if applicable, and the geometric means as well as the 95% CI were calculated. The accumulation of ridaforolimus was assessed for C_max_ and for AUC_0-24hr_ (geometric mean ratio Day 19 / Day 1 and 90% CI).

### Statistical analysis

In accordance with Chinese State Food and Drug Administration guidelines, at least 8–12 evaluable patients should be recruited. It was estimated that 15–16 patients were required to ensure that PK data would be obtained from at least 12 evaluable patients. The PK data and safety profiles were analyzed on an intent-to-treat basis and summarized descriptively.

## Results

### Patient characteristics

Between July 14, 2011 and September 29, 2011, sixteen patients were enrolled. Fifteen received ridaforolimus, one patient withdrew consent before administration of ridaforolimus. Patient characteristics are summarized in Table 1. The median age of patients was 51.3 years (range, 27–71); 80% of the patients had an ECOG performance status 1 and the rest had a status of 0. The most common tumor types were colon and breast cancer (33% and 20% respectively). Eight patients discontinued treatment because of progressive disease, six patients discontinued because of AEs, and one patient was lost to follow-up.

**Table 1:** Baseline characteristics of participants

**Characteristics**	**N = 15**
Median age (range, years)	51.3 (27–71)
Age group, years, n (%)	
49 and Under	7 (46.7)
50 to 59	4 (26.7)
60 and Above	4 (26.7)
Gender n (%)	
Male	5 (33.3)
Female	10 (66.7)
ECOG performance status n (%)	
0	3 (20)
1	12 (80)
Tumor type, n (%)	
Esophageal cancer	1 (6.7)
Gastric cancer	2 (13.3)
Colon cancer	5 (33.3)
Rectum cancer	2 (13.3)
Breast cancer	3 (20.0)
Soft tissue sarcoma	2 (13.3)

### Pharmacokinetic analysis

**Figure 1:**
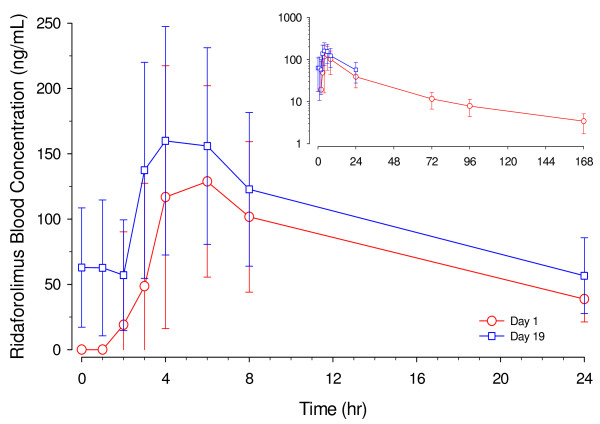
Blood concentration profiles (arithmetic mean +/− SD) of ridaforolimus following administration of a single oral dose or multiple once daily dosages of 40 mg of ridaforolimus in Chinese patients with advances or metastatic solid tumors (Day 1 n = 15 and Day 19 n = 13).

Figure 1 shows the time profiles of whole blood concentrations of ridaforolimus on Day 1 (post single dose) and Day 19 (post multiple dosages). Table 2 summarizes the PK parameters of ridaforolimus in whole blood.

**Table 2:** Pharmacokinetic parameters for ridaforolimus following a single oral dose or multiple once daily oral dosages of 40 mg of ridaforolimus in Chinese patients with advanced or metastatic solid tumors

**Pharmacokinetic parameter**	**Ridaforolimus**		
	**N**	**Mean**^‡^	**95% CI**^‡^
Day 1*			
T_lag_ (hr)	15	2.00	(0.97, 8.07)
AUC_0-24_ (ng.hr/ml)^†^	13	1846	(1361, 2503)
AUC_0-∞_ (ng.hr/ml)	13	3648	(3044, 4372)
C_max_ (ng/ml)^†^	13	210	(150, 295)
C_24hr_ (ng/ml)^†^	15	34.8	(26.0, 46.8)
T_max_ (hr)	13	4.03	(2.00, 7.97)
Apparent t_1/2_ (hr)	15	52.9	(40.2, 65.6)
Day 19**			
AUC_0-24_ (ng.hr/ml)^†^	13	2014	(1485, 2731)
C_max_ (ng/ml)^†^	13	167	(119, 234)
C_24hr_ (ng/ml)^†^	13	49.7	(36.4, 67.7)
T_max_ (hr)	13	4.00	(0.00, 6.00)

^†^Back-transformed least-squares mean and confidence interval from a linear mixed-effect model, containing a fixed effect for day and a random effect for subject, performed on natural log-transformed values.

^‡^Geometric Mean and back-transformed 95% CI for AUC_0-24hr_, AUC_0-∞_, C_max_, and C_24hr_. Harmonic Mean and jackknife SD for T1/2. Median and Range (Min, Max) for T_lag_ and T_max_.

^*^Both for Day 1 and Day 19 no samples were taken between 8 hr and 24 hr post dose. However, for some patients no concentration levels above the lower limit of quantification could be obtained until 8 hr post dose on Day 1. Consequently some PK parameters could not be calculated for these patients. In addition, Tlag was presented for all patients on Day 1.

^**^Two subjects were not evaluable for PK on Day 19 due to AE-related dose reduction.

After a single oral dose of ridaforolimus on Day 1, quantifiable drug levels were observed after a median absorption lag time of 2.00 h (T_lag_, range: 0.97–8.07 h). Peak concentrations occurred after a median time of 4.03 h (T_max_, range: 2.00–7.97 h). The geometric mean AUC_0-∞_ was 3648 ng · h/mL (CV 34.3%). The elimination half-life (t_½_) was 52.9 h (CV 24%). There was large between-patient variability see (Figure 1).

The geometric mean values for C_max_ were 210 and 167 ng/mL and median values were 192 and 203 ng/mL on Day 1 and Day 19, respectively. The geometric mean AUC_0-24hr_ was 1846 and 2014 ng · h/mL on Day 1 and Day 19, respectively. The accumulation ratio for Day 19 (steady state) / C_max_ on Day 1 (single dose) was 1.1 (90% CI 0.8–1.4) for C_max_ and 1.3 (90% CI 1.0–1.6) for AUC_0-24hr_.

### Safety evaluation

A once a day oral dose of 40 mg of ridaforolimus was generally well tolerated. Four patients (26.7%) developed serious AEs. All four serious adverse events were considered as a result of progression of malignant disease, and were not considered drug-related by the investigator. Two of them died from uncontrollable plural effusion, one from obstructive jaundice. Another patient developed apnea caused by the collapse of the tracheal which was infiltrated massively by fibrosarcoma. Six patients discontinued treatment as a result of AEs and five of these patients discontinued due to drug-related AEs: three as a result of drug-related interstitial lung disease, one as a result of drug-related fatigue, and another one as a result of drug-related stomatitis. One patient discontinued due to apnea cause by collapse of the tracheal which was defined as serious AE described previously.

A total of 135 non-serious AEs were reported by the fifteen patients; 111 of these AEs were deemed by the investigator as drug related. Table 3 lists the most commonly reported AEs (>20% incidence). Of the 135 non-serious AEs reported, 98 were considered to be Grade 1 or 2, 11 were considered to be Grade 3, and 2 were considered to be Grade 4 in severity. Grade 3/4 AEs are listed in Table 4.

**Table 3:** **Most common** (>**20**%) **non**-**serious adverse events**

**Adverse event**	**Number of patients and percentage**
Stomatitis	12, 80%
Proteinuria	11, 73.3%
White blood cell count decrease	8, 53.3%
Hyperglycaemia	7, 46.7%
Pyrexia	6, 40%
Gamma-glutamyltransferase increased	6, 40%
Anemia	5, 33.3%
Platelet count decrease	5, 33.3%
Hypoalbuminaemia	5, 33.3%
Blood cholesterol increased	5, 33.3%
Fatigue	4, 26.7%
Blood alkaline phosphatase increased	4, 26.7%
ALT increase	3, 20%
Interstitial lung disease	3, 20%

**Table 4:** **Grade 3**/**4 non**-**serious adverse events**

**Grade 3**	**Number of patients and percentage**
Anaemia	2/15, 13.3%
Stomatitis	2/15, 13.3%
Fatigue	2/15, 13.3%
Platelet count decrease	1/15, 6.7%
Constipation	1/15, 6.7%
Gamma-glutamyltransferase increase	1/15, 6.7%
Proteinuria	1/15, 6.7%
**Grade 4**	
Anaemia	1/15, 6.7%
Platelet count decrease	1/15, 6.7%

### Efficacy analysis

The mean duration of ridaforolimus treatment was 9.6 weeks (range, 0.2–22 weeks). Eleven patients had been evaluated for efficacy, all of them achieved stable disease as best efficacy, including one patient with esophageal cancer, two with gastric cancer, four with colon cancer, two with rectal cancer, one with breast cancer, and one with fibrosarcoma.

## Discussion

The primary objective of this phase I trial was to investigate the PK profiles as well as the tolerability of ridaforolimus in Chinese patients with advanced solid tumors. The daily oral dose of 40 mg of ridaforolimus was based on the results of previous phase I clinical studies, which investigated various dosing schedules in non-Chinese patients (internal data).

Although there was moderate-high variability between patients, our results show that, in general, ridaforolimus was slowly absorbed after oral administration. The AUC_0-24hr_ at steady state (Day 19, 2014 ng · h/mL) was lower than AUC_0-∞_ after single dose administration (Day 1, 3648 ng · h/mL), indicating that the blood PKs of ridaforolimus in our patients were non-linear. It is considered due to saturable binding of ridaforolimus to FK506 binding protein. With regard to drug interactions, all medications that were known to affect the CYP3A4 metabolism of ridaforolimus were discontinued before administration of ridaforolimus. Concomitant medications which may affect the pH of the stomach (e.g., antacids) are thought unlikely to have an effect on ridaforolimus PK due to drug solubility but could affect the dissolution of the enteric coated tablet.

The PK profiles of ridaforolimus in our group of Chinese patients did not differ from the PK data reported for Japanese [13] or Caucasian patients with advanced solid tumors from the internal PK data (Table 5).

**Table 5:** **Comparison of PK parameters between Chinese**, **Caucasian and Japanese patients**

**PK parameters**	**Chinese**	**Caucasian**	**Japanese**
Single dose			
AUC_0-24_ (ng.hr/ml)^a^	1846 (1361, 2503)	992(801, 1230)	1,470 (914, 2,360)
Cmax (ng/ml)^a^	210 (150, 295)	112(84.6, 148)	187 (115, 304)
Tmax (hr)^b^	4.03 (2.00, 7.97)	2.79(1.42, 9.78)	4.00 (4.00, 8.00)
t_1/2_ (hr)	52.9 (40.2, 65.6)^c^	42.0(17.7)^d^	55.8 (50.95, 60.65)^c^
Multiple dose			
AUC_0-24_ (ng.hr/ml)	2014 (1485, 2731)	1882(1630, 2180)	2,120 (1,320, 3,410)
C_max_ (ng/ml)	167 (119, 234)	160(136, 188)	188 (116, 306)
T_max_ (hr)	4.00 (0.00, 6.00)	--	4.04 (4.00, 8.15)
Accumulation ratio^e^			
AUC_0-24_	1.3(1.0, 1.6)	1.90(1.63, 2.21)	1.44 (1.05, 1.99)
C_max_	1.1(0.8, 1.4)	1.43(1.22, 1.68)	1.01 (0.674, 1.50)

^a^Geometric mean (95% confidence interval), ^b^Median (min, max), ^c^Harmonic mean (jack-knife SD), ^d^Harmonic mean (pseudo SD), ^e^Geometric mean ratio (90% confidence interval).

Amongst Chinese patients, the most common AE was stomatitis (12 patients, 80%), which was similar to the results of previous studies in non-Chinese populations. In our study, stomatitis occurred soon after initiation of ridaforolimus. In most patients, stomatitis could be alleviated by short treatment interruption. One patient discontinued the study due to stomatitis. Proteinuria (11 patients, 73.3%) was reported more often in this study than in non-Chinese populations [11-13]. Three patients developed Grade 2 interstitial pneumonitis, but all recovered after interruption of ridaforolimus and treatment with glucocorticoids. Mechanism-induced elevations in serum glucose, cholesterol and triglycerides are known to occur with mTOR inhibitors, including ridaforolimus [11-14]. Summary statistics for changes from baseline over time shown no clinically significant elevations in serum cholesterol, serum glucose, or serum triglycerides; however, an elevation in these three parameters was observed from baseline after ridaforolimus dosing in this study.

Eleven of 15 patients achieved stable of disease in this study. Although preliminary results are encouraging, the absence of partial or complete responses indicates that ridaforolimus might provide disease control rather than tumor shrinkage in the patients with chemo-refractory tumors. As reported before, objective response rates with rapamycin derivatives have been low in most tumor types [15,16]. A biomarker finding strategy could be considered as an effective way to select the possible candidates for ridaforolimus treatment. The identification of active status of the PI3K–AKT–mTOR pathway may be helpful to define the subgroups population potentially sensitive to mTOR inhibitors.

In conclusion, our results show that the overall PK and safety profile of ridaforolimus in Chinese patients with advanced solid tumors are generally consistent with that reported in non-Chinese patients with refractory or advanced solid tumors. The adverse effects of ridaforolimus were moderate and acceptable.

## Abbreviations

MTOR: Mammalian target of rapamycin; PK: Pharmacokinetic; AE: Adverse events; PFS: Progression free survival; ECOG: Eastern cooperative oncology group.

## Competing interests

The authors declare that they have no competing interests.

## Authors’ contributions

All authors participated in the drafting and editing of the manuscript. All authors read and approved the final manuscript.
